# Incremental Phonological Encoding during Unscripted Sentence Production

**DOI:** 10.3389/fpsyg.2012.00481

**Published:** 2012-11-09

**Authors:** T. Florian Jaeger, Katrina Furth, Caitlin Hilliard

**Affiliations:** ^1^Brain and Cognitive Sciences, University of RochesterRochester, NY, USA; ^2^Department of Computer Science, University of RochesterRochester, NY, USA; ^3^Program for Neuroscience, Boston UniversityBoston, MA, USA; ^4^Department of Psychology, University of IowaIowa, IA, USA

**Keywords:** sentence production, phonological encoding, phonological overlap, inhibition, unscripted speech

## Abstract

We investigate phonological encoding during unscripted sentence production, focusing on the effect of phonological overlap on phonological encoding. Previous work on this question has almost exclusively employed isolated word production or highly scripted multi-word production. These studies have led to conflicting results: some studies found that phonological overlap between two words facilitates phonological encoding, while others found inhibitory effects. One worry with many of these paradigms is that they involve processes that are not typical to everyday language use, which calls into question to what extent their findings speak to the architectures and mechanisms underlying language production. We present a paradigm to investigate the consequences of phonological overlap between words in a sentence while leaving speakers much of the lexical and structural choices typical in everyday language use. Adult native speakers of English described events in short video clips. We annotated the presence of disfluencies and the speech rate at various points throughout the sentence, as well as the constituent order. We find that phonological overlap has an inhibitory effect on phonological encoding. Specifically, if adjacent content words share their phonological onset (e.g., *hand the hammer*), they are preceded by production difficulty, as reflected in fluency and speech rate. We also find that this production difficulty affects speakers’ constituent order preferences during grammatical encoding. We discuss our results and previous works to isolate the properties of other paradigms that resulted in facilitatory or inhibitory results. The data from our paradigm also speak to questions about the scope of phonological planning in unscripted speech and as to whether phonological and grammatical encoding interact.

## Introduction

In order to communicate, speakers have to encode their intended message into a series of articulatory motor commands that create the continuous signal perceived by interlocutors. This process is broadly assumed to involve several stages of linguistic encoding (e.g., Garrett, 1980; Levelt, 1989; Bock and Levelt, 1994). Psycholinguistic research frequently distinguishes between lexical and grammatical processing. Grammatical encoding involves the assignment of functional roles (e.g., what is the subject of the sentence? What is the object?) and the sequential ordering of words as well as constituents. Lexical production involves the retrieval of the individual words’ lexico-syntactic and phonological information. Lexical production is often assumed to involve two – more or less separable – stages, lexical selection and phonological encoding (Dell, 1986; Levelt et al., 1999; Stemberger, 1985). Lexical selection begins with activation traveling from conceptual-level nodes to lemmas and ends with the selection of one, or potentially several, lemmas (sometimes also called L-level nodes, Goldrick, 2006). Lemmas are assumed to contain or be linked to lexico-syntactic information, such as subcategorization frames and other syntactic and argument structure requirements of a word. The second stage of lexical production, phonological encoding, ends with the selection of a phonological representation for the word, which forms the input to articulation.

Here, we are interested in phonological encoding during unscripted sentence production. In everyday contextualized language use, lexical and grammatical production take place simultaneously. Phonological encoding of words occurs at the same time as speakers choose between a large set of possible structures with the goal to produce well-formed utterances. One question that arises is how speakers manage to maintain relative fluency (only about 1 in 20 words of spontaneous speech is disfluent, Shriberg, 1996) while achieving speech rates of 3–5 syllables per second in conversational speech (e.g., Bell et al., 2003). Part of the answer is that the phonological encoding of several words takes place simultaneously, so that phonological encoding of neighboring words can overlap temporally (e.g., Dell and Reich, 1981; Griffin, 2003; Smith and Wheeldon, 2004).

The primary goal of this article is to develop and test a paradigm for the investigation of phonological encoding during unscripted sentence production. Specifically, we investigated the effects of phonological onset overlap between words in the same sentence (e.g., *Hannah handed the boy a hammer*). Competition models of phonological encoding predict that onset overlap between adjacent words inhibits their phonological encoding compared to sequences of phonologically unrelated words (Peterson et al., 1989; Sevald and Dell, 1994; O’Seaghdha and Marin, 2000). We use the terms inhibition and facilitation as convenient shorthands to refer to behavioral effects, such as a slow down/speed up of the speech rate or an increase/decrease in speech errors, without implying a specific mechanistic architecture.

Competition models have received support from studies that have found higher rates of speech errors, slower speech rates, and longer speech onset latencies in the presence of phonological onset overlap (e.g., Bock, 1987; Martin et al., 1989; Sevald and Dell, 1994; O’Seaghdha and Marin, 2000; Wheeldon, 2003). However, there are also numerous studies that have found facilitation of speech onset latencies in the presence of phonological onset overlap, as reflected in shorter speech onset latencies (e.g., Meyer, 1991; Meyer and Schriefers, 1991; Schriefers, 1999; Smith and Wheeldon, 2004; Schnur et al., 2006). There are reasons to believe that some of these apparently conflicting results are a consequence of the specific paradigm employed (for discussion, see Damian and Martin, 1999; Starreveld, 2000). We return to this point in the Section “Discussion.”

Most previous work on the effect of onset overlap on phonological encoding has investigated the production of isolated words in the absence of a sentence or discourse context (isolated word production; e.g., Meyer and Schriefers, 1991; Peterson and Savoy, 1998; O’Seaghdha and Marin, 2000; Jescheniak and Schriefers, 2001). To the extent that phonological encoding has been investigated in context, these investigations have usually involved language production that was *scripted* in at least two senses (but see Bock, 1987; Martin et al., 1989).

First, participants were typically asked to produce the same type of word (e.g., nouns) or the same types of sequences of words (e.g., color adjective followed by noun) in every trial. Even when more complex utterances are elicited, this is typically done by means of a carrier phrase that is repeated in every trial. For example, participants might have to produce sequences like “The X moves above/below the Y,” where X and Y are the only content words that change from trial to trial (e.g., Griffin, 2003; Smith and Wheeldon, 2004). Additionally, almost all investigations of phonological encoding in word sequences have been limited to the production of single phrases (typically noun phrases; for notable exceptions, see Bock, 1987; Rapp and Samuel, 2002; Smith and Wheeldon, 2004; Schnur et al., 2006). It is thus unclear to what extent these tasks reflect phonological encoding during everyday language use, where lexical and phonological production coincides temporally with grammatical encoding.

Second, many – though not all – of these paradigms involve orthographic comprehension temporally coinciding with the production of the target word (e.g., Meyer, 1991; Meyer and Schriefers, 1991; Jescheniak and Schriefers, 2001; Schnur et al., 2006; Janssen and Caramazza, 2009). This raises the question to what extent findings from the paradigms generalize to language production when no orthographic comprehension is involved.

Previous work on phonological encoding during unscripted speech has mostly employed speech error corpora (e.g., Shattuck-Hufnagel, 1979; Dell and Reich, 1981; Stemberger, 1990). These studies have found both perseverative and anticipatory speech errors. In perseverative speech errors, a phonological segment from a previous word interferes with the production of the current word (e.g., *beef noodle* → *beef needle*). In anticipatory speech errors, a segment of an upcoming word interferes with the production of the current word (e.g. *reading list* → *leading list*). Studies of naturally occurring speech errors thus provide evidence that the phonological encoding of neighboring words can overlap during unscripted speech. Speech error corpora provide ecologically valid evidence about phonological encoding during everyday contextualized language production (although other factors can limit their usability; see, e.g., Pérez et al., 2007).

Here, we aim to contribute a paradigm for the investigation of phonological encoding during lexically and structurally unscripted speech, while still allowing relatively direct control over the data researchers will obtain. Our specific goal is to investigate how phonological overlap between words in a sentence affects their phonological encoding during unscripted speech and how this in turn affects grammatical encoding. There is a striking lack of such studies. Out of close to 200 experiments on the effect of phonological onset overlap that we surveyed for this article, only two investigated unscripted speech, while still providing a high degree of control for the researcher (Bock, 1987; Martin et al., 1989).

The analyses we conduct also shed light on two additional questions. First, we can use the data we analyze below to investigate the scope of phonological encoding during unscripted speech. Second, the results presented below speak to recent claims that phonological encoding interacts with grammatical encoding (Abrams and Rodriguez, 2005; Janssen et al., 2008; Janssen and Caramazza, 2009). These claims are based on findings from scripted sentence production. In the Section “General Discussion,” we evaluate this claim against data from unscripted speech.

Our approach builds on the picture description paradigm developed by Bock (1987). Bock (1987) employed picture description to elicit transitive sentences, which can be realized as active (1a) or passive (1b). Before each picture, participants were primed by a noun that either shared its phonological onset with the agent expression or the theme expression of the target sentence. For example, the prime *mat* was used to affect phonological encoding of the agent *man* or the prime *beet* was used to affect phonological encoding of the theme *bee*.

**Table:** 

(1)	(a)	The bee stung the man.
	(b)	The man was stung by the bee.

Bock (1987) found that phonologically priming a word leads to an increased likelihood that the word would be mentioned later in the sentence. Put differently, speakers preferred syntactic structures that allowed them to delay the production of the phonologically primed word. This finding thus suggests that phonological overlap leads to inhibition.

Here, we investigate the effects of phonological overlap in unscripted speech when the both the “prime” and the “target” are produced as part of an unscripted sentence (e.g., *Hannah handed the ice cream to the boy*). As a consequence, any “prime” in our paradigm is also a “target” (and vice versa) – just as in everyday language use.

To this end, we investigate effects of phonological overlap within a sentence on phonological and grammatical encoding. Specifically, we investigate both anticipatory and perseverative effects of phonological onset overlap on fluency, speech rate, and constituent order. Anticipatory effects occur before or during the production of the first word of a pair of phonologically overlapping word (e.g., before or during the production of *Hannah* in *Hannah handed the ice cream to the boy*). Perseverative effects occur after the first and before the second word of the pair (e.g., between *Hannah* and *handed* in the same example). Our studies focus exclusively on onset overlap (e.g., *hand-hammer*), as opposed to rhyme overlap (e.g., *lock-sock*)^1^.

The data for the current study came from an experiment reported in Jaeger et al. (2012). In Jaeger et al. (2012), we found that speakers exhibit a bias against sequences of words with overlapping phonological onsets. Specifically, speakers avoided such sequences when alternative lexicalizations for the same meaning were available. This suggests that phonological properties can affect even lexical selection and, hence, processes that precede phonological encoding. We also found preliminary evidence that phonological overlap affects fluency. Here, we extend these investigations. We added several layers of annotation to the database from Jaeger et al. (2012), in order to analyze constituent order, speech onset latencies, speech rate, and the distribution of overt disfluencies *throughout the sentence*. Next we describe this database. Following that, we spell out our predictions.

## Overview of the Database

Following Bock (1987), we employed scene description to investigate the effects of phonological overlap on unscripted sentence production (here based on videos, rather than pictures). Another similarity with the experiments reported by Bock is that we employed a word alternation to investigate potential effects of phonological encoding on constituent order and hence grammatical encoding (we used the ditransitive alternation; Bock investigated active/passive and conjunct ordering). In the dative or ditransitive alternation in (2), the post-verbal theme can be ordered theme-first (a) or theme-last (b).

**Table:** 

(2)	(a)	Hannah gave [_NP_ a pan] [_PP_ to the woman]. [Theme-first order]
	(b)	Hannah gave [_NP_ the woman] [_NP_ a pan]. [Theme-last order]

In addition to some of the minor differences already mentioned, the design of our experiment differs from the design employed by Bock in one important aspect. In the experiments presented in Bock (1987), participants were primed by a noun that either shared its phonological onset with one of the arguments in the target sentence. One goal of the current work is to move further toward a paradigm that enables the investigation of production-internal effects of unscripted language production. Hence, instead of employing isolated primes, videos were designed to elicit sentences with and without phonological overlap between words *within the same sentence*.

In the crucial phase of the experiment, participants watched short 3D-animated videos that displayed physical transfer events. Participants were instructed to produce past tense sentences, which pilot experiments had shown to be the preferred tense (presumably because videos were described after they had played). All target events were compatible with ditransitive descriptions involving any of the three verb lemmas *give*, *hand*, or *pass*. Physical transfer events described by these verbs involve an agent (the person transferring something), a theme (the thing being transferred), and a recipient (the person receiving the thing being transferred). All of the three verbs participate in the ditransitive alternation, as in (2) above.

### Materials and methods

#### Design

**Figure 1:**
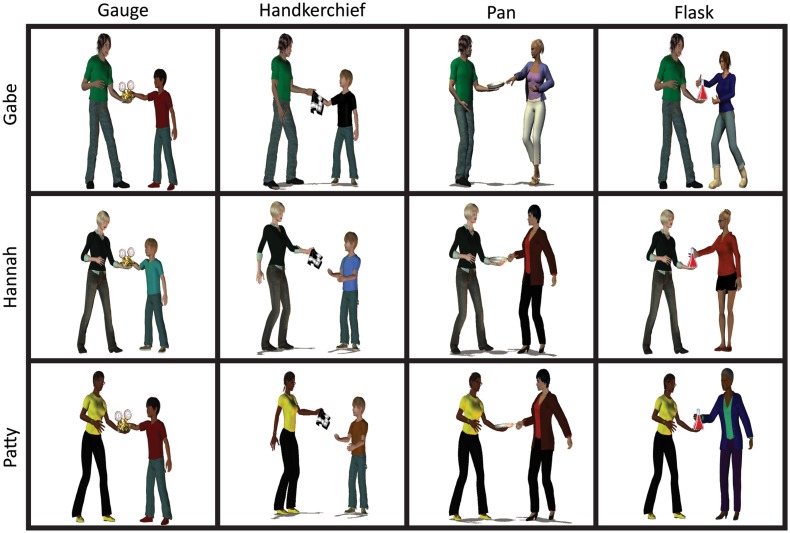
**Illustration of design**. Reprinted with permission from Jaeger et al. (2012).

The phonological form of both the subject and the theme expression was manipulated to form a 3 × 4 design. In target videos, subject expressions were *Gabe*, *Hannah*, or *Patty*, so that their onset overlapped with one of six possible verb forms, corresponding to the three verb lemmas (*give*, *hand*, and *pass*). Most participants predominantly produced the past tense forms (*gave*, *handed*, and *passed*), but a few participants produced present tense forms (*gives*, *hands*, and *passes*). Theme expression either did or did not overlap with one of the verb forms (i.e., the theme expression’s onset was /g/, /h/, /p/, or different; for details, Jaeger et al., 2012). Recipient expressions never overlapped with any of the three verbs. Recipients were designed to elicit the labels *boy* and *woman*. Figure 1 illustrates the design.

#### Procedure

Consistent naming of the subject and theme expressions, which is crucial for our manipulation of phonological overlap, was achieved by a disguise memory task. In the first phase of the experiment, participants were exposed to four characters and 40 objects that they were instructed to remember. These characters and objects form the exhaustive set of subject and theme expressions for the crucial phase of the experiment. The familiarization phase was followed by a test phase that ensured that participants had reached sufficiently high performance on the names for both characters and objects. For further detail on the first two phases of the experiment, refer to Jaeger et al. (2012).

The third phase of the experiment was the crucial one in which participants described videos. Participants had been instructed that this phase was meant to help them remember the names for characters and objects by repeatedly using them in sentences. Participants also were instructed that there would be a fourth test phase after the video descriptions, which never took place. After the experiments, participants were debriefed and informed about the true purpose of the experiment.

Each list consisted of 54 target videos and 72 filler videos for a total of 126 videos. Filler videos involved actions not described by any of the three target verbs. Fillers contained the three subject characters from the target videos (*Gabe*, *Hannah*, and *Patty*) as well as fourth character (*Simon*). Theme objects also occurred in the fillers. Across fillers and items, each of the four subject characters and each of the objects occurred approximately equally often (± one occurrence). Each list was divided into three blocks of 18 target videos and 24 filler videos each. Each block formed a Latin square design. Across all three blocks, participants saw each item in all its conditions. That is, if in block 1, a theme referent occurred with the subject referent Hannah, in blocks 2 and 3 that same theme referent occurred with the other subject referents, Gabe and Patty, respectively. One or two filler trials intervened between each target video. The order of presentation for the fillers was randomly determined. Each participant was assigned randomly to one of three lists that together counterbalanced the order of the three experimental blocks. The analysis reported below collapsed over all three blocks, since this never changed results.

#### Annotation

Three undergraduate research assistants transcribed 2970 target trials collected at the University of Rochester from 55 undergraduate students. For the analyses reported in Jaeger et al. (2012), we marked all cases with errors, included (i) wrong labels to refer to the subject, theme, or recipient, (ii) use of a verb lemma different from *give*, *hand*, or *pass*, or (iii) failure to produce a ditransitive structure. The error annotation revealed that some participants had produced present tense rather than past tense forms. Since present tense form accounted for about a third of the data, we decided to include them in the analysis. After exclusion of all other errors, this left 2594 cases. After excluding four participants with error rates of 50% or more, 2498 data points from 51 participants remained.

Here, we conducted additional annotation to remove cases, in which participants (i) failed to correctly recall the intended labels for the subject or theme expression (four cases), (ii) explicitly struggled recalling that label (nine cases), (iii) wrongly labeled the recipient with one of the names of the three subject characters (nine cases), (iv) started the sentence with the verb rather than the subject (nine cases), (v) used pronominal recipient labels (five cases), or used the label *girl*, instead of the intended *woman*, to refer to the recipient, which overlapped with one of the potential verbs (30 cases)^2^.

We then added three new layers of annotation to the remaining 2430 cases from 51 participants (each of which had exclusion rates below 50%): constituent order, disfluency, and speech rate (including speech onset latencies). Annotations were conducted by the same research assistants who had provided the annotations for Jaeger et al. (2012; see acknowledgments). The annotators were blind to the experimental hypotheses. We describe each annotation as part of the studies reported below.

#### Overview of analyses

Below, we begin by examining the effects of phonological overlap on speakers’ constituent order preferences (theme-last vs. theme-first) and the distribution of disfluency and changes to the speech rate throughout the sentence depending on whether and where the sentence contained words that shared their phonological onset. For all three of these behavioral measures, we ask whether phonological overlap inhibited or facilitated phonological – and, as a consequence, grammatical encoding.

The paradigm employed here provides only a limited degree of control to the experimenter. For example, while the phonology of the subject and theme expression was controlled via the characters and objects in the scene, participants were free to choose whatever verbs they preferred to describe the scene. As a consequence, the scene descriptions in the database fall into five conditions with regard to what parts of the sentence exhibit phonological overlap. These five conditions are summarized in Table 1. Below we focus on the 2061 cases that correspond to conditions (1–4) in Table 1. The four conditions form an (unbalanced) 2-by-2 design, crossing phonological overlap between the subject and verb (subject-verb overlap) with phonological overlap between the verb and object (verb-object overlap).

**Table 1:** **Types of phonological overlap observed in the data**.

Phonological overlap condition	Cases		Example
Subject-verb (only)	575	(24%)	*Hannah handed the teapot to a boy*
Verb-theme (only)	352	(14%)	*Patty handed the hammer to the boy*
Subject-verb-theme	175	(7%)	*Hannah handed the hammer to the boy*
None	959	(39%)	*Patty handed the guitar to the boy*
Subject-theme (only)	369	(15%)	*Patty handed the pan to the boy*

We excluded cases in which the subject and the theme overlapped phonologically without either of them overlapping with the verb (subject-theme overlap). The reason for this is threefold. First, in the 2-by-2 design, these cases would be grouped together with the no-overlap condition (row 4 in Table 1). This would be undesirable since it would mix cases without phonological overlap and cases with phonological overlap. Second, although the statistical methods we describe below theoretically would have allowed us to analyze all five conditions simultaneously, the unbalanced nature of the data makes this analysis difficult to present^3^. Third, the information gain of the more complex analysis would have been minimal.

## Constituent Order

We begin by trying to replicate the results of Bock (1987). In Bock’s experiments participants heard and repeated a prime and then described a picture. Pictures either elicited simple transitive sentences (e.g., *The bee stung the man* or *The man was stung by the bee*) or noun phrase coordination (e.g., *A woman carrying a lamp and a plant* vs. … *a plant and a lamp*). Picture description contained one word that had the same phonological onset as the prime (e.g., either *man* or *bee* was phonologically primed for the example transitive sentence). Bock found that speakers were more likely to order words later in the sentence when they overlapped phonologically with the prime.

Bock (1987) attributes this effect to availability-based production, a preference to produce material that is readily available earlier whenever grammar permits so. Availability-based effects are well-documented for constituent order preferences and beyond (e.g., Levelt and Maassen, 1981; Ferreira, 1996; Ferreira and Dell, 2000; Prat-Sala and Branigan, 2000; Branigan et al., 2008; for an overview, see Jaeger and Norcliffe, 2009).

The assumption that phonological overlap leads to inhibition of phonological encoding has, however, received mixed support. On the one hand, several studies have found slower speech rates and longer durations for words that share their phonological onset with recently produced words (Sevald and Dell, 1994; O’Seaghdha and Marin, 2000; Wheeldon, 2003). On the other hand, a large number of studies on isolated word production have found facilitatory effects of phonological overlap (e.g., Meyer and Schriefers, 1991; Peterson and Savoy, 1998; O’Seaghdha and Marin, 2000; Jescheniak and Schriefers, 2001).

If phonological overlap inhibits phonological encoding in unscripted speech, we expect verb-theme overlap to lead to an increased preference for theme-last constituent orders.

### Materials and methods

#### Annotation

The three verb lemmas exhibited their expected subcategorization bias: *give* and *gave* were roughly equi-biased (NPNP: 45%, NPPP: 55%) and so was *hand* (NPNP: 55%, NPPP: 45%). The verb *pass* exhibited a strong preference for the NPPP order (NPNP: 28%, NPPP: 72%). Overall, this resulted in equal proportions of NPNP and NPPP orders (50% each). Based on 100 blindly sampled cases, inter-annotator agreement was perfect (Kappa = 1.0).

#### Analysis

Paradigms that elicit unscripted speech, such as the one employed here, typically result in highly unbalanced data. The analysis of unbalanced data requires different statistical methods than standardly employed in the analysis of balanced behavioral data from psycholinguistic experiments. Here we employed a logit mixed model analysis (Breslow and Clayton, 1993; for introductions, see Jaeger, 2008; for discussions of the particular benefits of these models for the analysis of unbalanced data sets, see also Jaeger, 2006, 2011; Bresnan et al., 2007).

Unscripted speech data also typically are both lexically and structurally heterogeneous compared to scripted productions. On the one hand, this is desirable: the increased heterogeneity of the data reflects the fact that everyday language is typically not restricted to a few sentence structures and a vocabulary in the double digits (unlike many language production experiments). But with the increased heterogeneity also come additional statistical challenges. In particular, the potential for confounds arguably increases. Less flexible (e.g., scripted) paradigms allow researchers to balance potential confounds across the conditions of interest *prior to the experiment*. Although it should be kept in mind that researchers rarely exercise this advantage to the fullest extent – psycholinguistic experiments typically do not balance *all* known confounds prior to the experiment – paradigms like the one employed here do not offer the same degree of “control by design.” For those reasons, it is crucial to consider potential confounds during the statistical analyses.

In an effort to ensure replicability of our results, we considered a large number of potential confounds. These included the syllable frequency, word frequency, and neighborhood density for the (lexical head of the) subject, verb, theme, and recipient expressions as well as two collocational control predictors, capturing the probability of co-occurrences of the subject and verb and the co-occurrence of the verb and object. We also annotated whether the subject, verb, theme, and recipient expression had been repeated from the most recent target trial and whether the syntactic structure had been repeated. Finally, we obtained plausibility ratings for all sentences. A full list of control predictors, how they were obtained, and their statistics across the conditions of interest are given in the Section “Overview over Control Predictors” in the Appendix.

A first round of analyses with these control predictors (not reported here) confirmed the expected control effects. For example, we found highly significant syntactic priming effects on constituent order (Bock, 1986) and this effect interacted with verb repetition, as observed in previous studies (Pickering and Branigan, 1998). Here we report simplified results. We employed principal component analysis to both effectively and efficiently control for potential confounds in our analysis of phonological overlap effects. This procedure is described in the Section “Controlling for Potential Confounds” in the Appendix. Principal components of all potential confounds were derived. The most influential of these principal components were then entered into the analysis of constituent order, thereby ruling out confounds while assessing the effect of phonological overlap on constituent order. Below we simply report the results of interest (i.e., the effect of phonological overlap between the subject, verb, and theme).

Mixed effect analyses also require specification of a random effect structure. Preferably, both random intercept and slopes should be specified for any relevant grouping variable. Here, the most relevant grouping variable is participants (like in other corpus-based work, it is not clear what would constitute an item). Section “Details on the Random Effect Structure of the Analyses” in the Appendix describes the procedure that we employed to determine the random effect structures for the analyses reported below.

Both the procedure used to control for potential confounds (see Controlling for Potential Confounds in the Appendix) and the procedure to determine the random effect structure (see Details on the Random Effect Structure of the Analyses in the Appendix) balanced consideration about power, overfitting, and the desire for a conservative analysis that is likely to replicate. In order to capture effects of the subcategorization bias of the different verbs, we also included a random intercept by verb form. We note that the qualitative results reported below did *not* depend on the choice of the random effect structure. They did, however, in some cases depend on the adequate control of confounds. Here and below, there were no signs of collinearity in any of the models unless otherwise mentioned (fixed effect correlations *r*s < 0.2).

Next, we present the results of a mixed logit model predicting speakers’ preference for theme-last over theme-first order dependent on subject-verb overlap, verb-theme overlap, and their interaction. All analyses reported below were conducted using the library *lme4* (release 0.999375–42, Bates et al., 2011) statistics software R (release 2.14.1, R Development Core Team, 2011). Plots were created with the library *ggplot2* (release 0.9.0, Wickham, 2009).

### Results

Participants were significantly more likely to produce theme-last sentences when the subject expression overlapped phonologically with the verb ($\hat{\beta }$ = 0.36, *z* = 2.6, *p* < 0.01). The effect of subject-theme overlap did not reach significance (*p* > 0.2) and neither did the interaction (*p* > 0.8).

**Figure 2:**
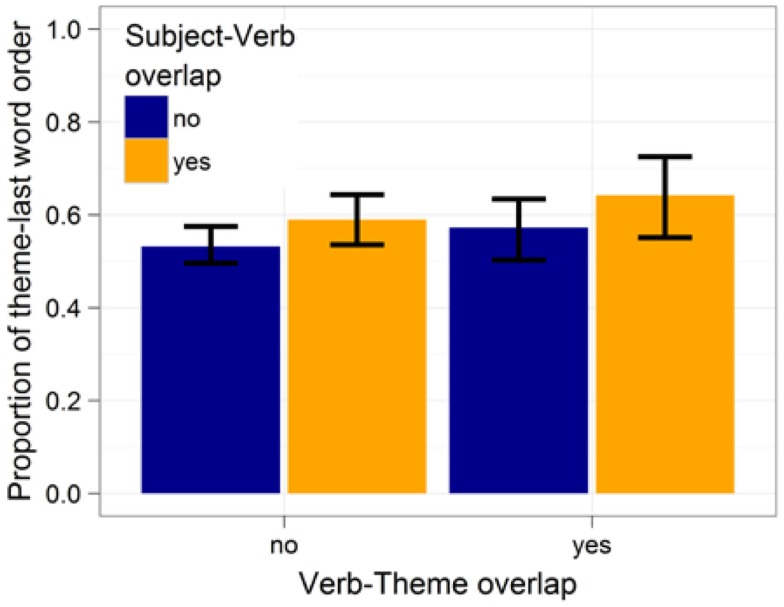
**Effects of subject-verb and verb-theme overlap on constituent order preferences for participants that produce each of the two possible constituent orders at least 10% of the time**. Proportions are out of 1256 cases. Error bars give 95% confidence interval.

Further examination of the data revealed that the lack of an effect for verb-theme overlap was due to many speakers producing one constituent order 100% of the time. When the analysis is restricted to speakers who produce each structure at least 10% of the time (i.e., at least 1–2 times), we find a significant inhibitory effect of verb-theme overlap $\hat{\beta }$ = 0.37, *z* = 2.2, *p* < 0.03. When the exclusion criterion was made more stringent (e.g., each structure having to be produced at least 15% of the time), this effect increases in size, suggesting that participants who allowed both theme-first and theme-last order preferred to place phonologically overlapping themes later in the sentence. For all of these auxiliary analyses, the effect of subject-verb overlap remained significant or marginally significant in the same direction as reported above (*p*s < 0.07 or smaller) and the interaction never reached significance (*p*s > 0.8). Figure 2 shows these effects for constituent order-varying participants. The effect of subject-theme overlap remained non-significant when only constituent order-varying participants were included in the analysis.

### Discussion

Previous work has found that speakers prefer to postpone the production of the theme (resulting in theme-last order) when the theme overlaps phonologically with a noun prime produced immediately before the sentence (Bock, 1987). Here too we found evidence of an inhibitory effect of phonological onset overlap on phonological encoding, as reflected in a preference to postpone the production of themes that share their phonological onset with the verb.

The effect size we found is quite comparable to that reported in Bock (1987). Translated into log-odds, Bock found effect sizes of 0.18 < $\hat{\beta }$ < 0.42 (cf. Bock, 1987, Figure 2). We found an effect size of $\hat{\beta }$ = 0.37 for verb-theme overlap. However, unlike in Bock’s experiments, the effect of verb-theme overlap emerged as significant only when participants who always produced the same constituent order were excluded from the analysis. One reason for this might be the slightly smaller number of participants in the current study (51 vs. 60). Another reason might be that Bock compared the effect of phonological priming of one constituent against phonological priming of another constituent. For the current stimuli, this would correspond to comparing the effects of phonological overlap between the verb and theme to the effects of overlap between the verb and the recipient. In the current experiment verb-theme overlap was compared only against cases without overlap.

The observed marginal effect of subject-verb overlap was not predicted. It is unclear why phonological overlap between the subject and verb affected the constituent order following the verb. For phonological overlap between the verb and the object, postponing the production of the object can presumably prevent suspension of speech due to difficulty in producing the object due. The same reasoning does not, however, apply to subject-verb overlap since it is unclear how postponing the theme provides further time for the production of the subject or verb. Note that there was no evidence that the effect of subject-verb overlap might be due to increased interference during the encoding of the theme. Such an effect should have resulted in an interaction between subject-verb and verb-theme overlap.

One explanation for the effect of subject-verb overlap is that the theme-last order is the “default” constituent order. Under the additional assumption that speakers chose between the different constituent orders some time before or during the articulation of the verb (cf. Kuperman and Bresnan, 2012), it is possible that speakers are more likely to go with the default order when they experience production difficulty during the verb.

Taken together with the results obtained by Bock (1987), the current findings seem to suggest that phonological onset overlap during unscripted language production results in inhibited phonological encoding. This would suggest that the facilitatory effects of phonological overlap observed in previous scripted multi-word studies are due to properties of the paradigm (e.g., Smith and Wheeldon, 2004; Janssen et al., 2008; Janssen and Caramazza, 2009). However, the evidence for inhibition reported so far is only indirect. So far, we have followed Bock in assuming that the preference for theme-last order for sentences with verb-theme overlap stems from production difficulty experienced when phonologically overlapping words are produced in close temporal proximity. This assumption can be tested more directly by examining whether phonological onset overlap indeed causes production difficulty. This is the purpose of the two studies reported next. We investigate the distribution of disfluencies and changes in the speech rate throughout the sentence depending on the absence or presence of phonological overlap.

## Disfluency

If the increased preference for theme-last order in the presence of phonological overlap between the verb and theme is indeed due to production difficulty caused by the phonological overlap, it should be possible to detect effects of phonological overlap on fluency. Production difficulty with upcoming material is typically reflected in an increased probability of hesitations, filled pauses, and restarts before the point of difficulty (Shriberg and Stolcke, 1996; Fox Tree and Clark, 1997; Clark and Wasow, 1998; Clark and Fox Tree, 2002). Thus, we expect to find increased disfluency before words that share their onset with recently produced words. To highlight parallels to work on speech errors, we will refer to this as *perseverative* effects of phonological overlap (cf. perseverative speech errors, Dell and Reich, 1981).

In our data, the subject always immediately preceded the verb. Hence, subject-verb overlap is expected to lead to increased production difficulty in the pre-verb region. The prediction for verb-theme overlap is somewhat more complicated. In the theme-first order, only one function word intervened between the verb and the theme expression in most of the target trials (usually the determiner *the*). In the theme-last order, however, the recipient phrase intervened between the verb and theme. Since the results from our constituent order analysis and previous work suggest that the effect of phonological overlap decreases with increasing distance between the overlapping words, we might expect effects of verb-theme overlap only in the theme-first order. It is, however, unclear whether the eventual constituent order affects when phonological encoding of the theme begins. According to availability-based production accounts, we would expect that speakers at least to some extent plan both the theme and the recipient in parallel (and then articulate whichever is ready for articulation first, cf. the Principle of Immediate Mention, Ferreira and Dell, 2000).

In addition to perseverative effects, it is possible that there are *anticipatory* effects immediately before the first of two phonologically overlapping words. Predictions for anticipatory effects depend on assumptions about the scope of phonological planning during incremental sentence production. This question is still subject of much debate, with some studies arguing that phonological encoding is radically incremental, proceeding at a scope of only one word at a time (Wheeldon and Lahiri, 1997; Griffin, 2003), while other studies suggest that several words are being planned in parallel. There is, however, broad agreement that there must be *some* overlap between the phonological encoding of adjacent words in spontaneous speech (e.g., to account for anticipatory phonological speech errors, Dell and Reich, 1981). Hence, if two words are sufficiently close to each other so that their phonological encoding overlaps, we expect to find anticipatory effects of phonological overlap on fluency.

Specifically, if phonological encoding of the verb starts before articulation of the subject, subject-verb overlap should also lead to increased disfluency in the pre-subject region. Similarly, verb-theme overlap might lead to increased disfluency in the pre-verb region if the phonological encoding of themes begins before articulation of the verb.

### Materials and methods

#### Annotation

**Figure 3:**
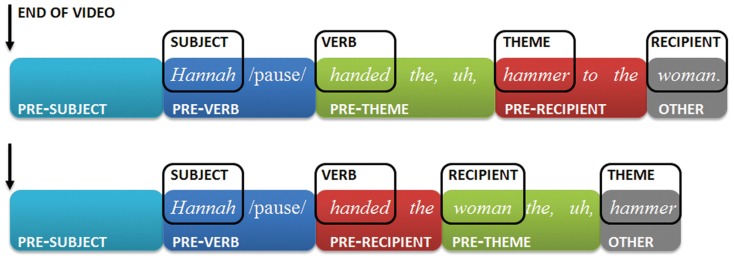
**Example sentence in theme-first (top) and theme-last order (bottom)**. The four annotation regions (pre-subject, pre-verb, pre-theme, and pre-recipient) are illustrated by colored boxes.

To facilitate a fine-grained investigation of localized production difficulty, the presence of pauses (suspension of speech), filled pauses (e.g., *uh*; *um*; *I mean*) and restarts (e.g., *ha-handed*; *the*, *the*, *hammer*) was marked for each of the four regions shown in Figure 3: before the subject expression’s head noun (henceforth the pre-subject region), before the verb (pre-verb), before the head noun of the theme expression (pre-theme), and before the head noun of the recipient expression (pre-recipient).

Restarts of a word (e.g., *Hann-Hannah*) or speech errors on a word (e.g., *Gave gave the*, *I mean*, *Gabe gave* …) were counted as disfluency preceding that word (i.e., the examples in parentheses were annotated as pre-subject disfluencies). Repetitions of a fully and successfully pronounced word, however, were counted as disfluency for the region following the word. For example, *Patty Patty passed* … was counted as a pre-verb disfluency.

It is possible that some of these latter cases are the result of barely audible (e.g., sub-phonemic) speech errors that the speaker seeks to correct. As a result, the analyses might underestimate effects on earlier sentence regions (counting these cases as disfluencies for both regions would inflate the Type I error rate of our analyses). Of all trials, 3.4% (80 cases) contained a pre-subject disfluency, 4.1% (83 cases) contained a pre-verb disfluency, 9.5% (192 cases) contained a pre-theme disfluency, and 9.1% (186 cases) contained a pre-recipient disfluency. Based on 100 blindly sampled cases, inter-annotator agreement was high (with Kappas ranging from 0.86 to 1.0 for the four sentence regions).

#### Analysis

To test these predictions, we conducted separate mixed logit analyses predicting the presence of disfluencies for each of the four sentence regions described above in Figure 3. The statistical procedure was identical to that described in the previous section (see Controlling for Potential Confounds in the Appendix). See the Section “Details on the Random Effect Structure of the Analyses” in the Appendix for details about the random effect structure. Results reported below did not depend on the choice of the random effect structure.

### Results

**Figure 4:**
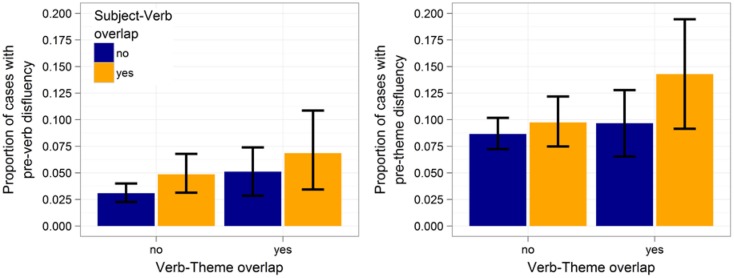
**Effects of subject-verb and verb-theme overlap on the likelihood of disfluencies in the pre-verb region (left) and pre-theme region (right)**. Proportions are out of 2061 cases. Error bars give 95% non-parametric confidence interval (obtained via bootstrap).

Table 2 summarizes the results. For the pre-subject region, we found no effect of subject-verb overlap (*p* > 0.7) or verb-theme overlap (*p* > 0.6). For the pre-verb region, both subject-verb and verb-theme overlap resulted in significantly more disfluencies (*p*s ≤ 0.05). These two effects are illustrated in Figure 4 (left). None of the main effects in any of the other regions reached significance, although the effect of subject-verb overlap approached marginal significance for the pre-theme region (cf. Figure 4, right). The interaction between subject-verb and verb-theme overlap never reached significance (*p*s > 0.2).

**Table 2:** **Disfluency results by sentence region**.

Phonological overlap	Subject-verb			Verb-theme			Subject-verb: verb-theme		
Region	$\hat{\beta }$	*Z*	*p*	$\hat{\beta }$	*z*	*p*	$\hat{\beta }$	*z*	*p*
Pre-subject	0.10	0.3	>0.7	−0.14	−0.4	>0.6	−0.01	−0.1	>0.9
Pre-verb	**0.67**	**2.7**	**<0.01**	**0.61**	**2.3**	**<0.02**	−0.22	−0.5	>0.6
Pre-theme	**0.39**	**2.4**	**<0.02**	**0.39**	**0.2.2**	**<0.04**	0.33	0.9	>0.3
Pre-recipient	−0.01	−0.1	>0.9	0.01	0.1	>0.9	0.38	1.0	>0.3

*Bold indicates the significant effects*.

### Discussion

Overall, we found converging evidence that phonological overlap inhibits lexical production: all significant effects were inhibitory in that disfluencies were more likely in regions that contained words that overlapped phonologically with other words in the sentence. This provides further support that phonological onset overlap during unscripted speech leads to production difficulty, as hypothesized by Bock (1987).

The predicted perseverative effects of phonological overlap were found in the pre-verb and pre-theme region. We also observed an anticipatory effect of verb-theme overlap in the predicted direction in the pre-verb region. No anticipatory effect of subject-verb overlap was observed in the pre-subject region.

No effects of phonological overlap were observed before the subject. The absence of an effect of subject-verb overlaps is somewhat surprising since earlier work suggests that speakers plan at least the subject and verb before initiating speech (Lindsley, 1975). One possibility is that this advance planning does not include phonological encoding of the verb, in which case no effect of subject-verb overlap is expected. Some evidence from studies on incremental planning of sequences of words suggests that speakers can initiate speech with advance phonological encoding of only one syllable (Griffin, 2003). The exact scope of phonological planning processes during sentence production is, however, still under debate (e.g., Wheeldon and Lahiri, 1997; Wheeldon, 2003). In particular, we know of no study that investigates this question for unscripted speech.

Another possible explanation for the lack of an effect in the pre-subject region is that when speakers experience production difficulty before the subject they simply delay speech onset. In our experiment, the subject expressions were always proper names. Thus speakers did not have the option of repeating readily available phrase-initial material (*the*, *the*, *the hammer*). Hence, speakers were left with two options: to produce a filled pause (*uh*, *um*) or to delay the speech onset. It is possible that most speakers preferred the latter option. If so, we would expect to observe an effect of subject-verb overlap on speech onset latencies. We test this prediction in the next section. Preliminary support for this hypothesis comes from the observation that pre-subject disfluencies were overall less likely, compared to all other sentence regions (pre-subject disfluencies: 3.4%, pre-verb: 4.1%, pre-theme: 9.5%, pre-recipient: 9.1%).

Since it is possible that effects of phonological overlap depend on the proximity of the phonologically overlapping words, we conducted additional *post hoc* analyses in which constituent order (theme-last vs. theme-first) was entered into the model along with its interactions with subject-verb overlap, verb-theme overlap, and their interaction. These analyses are reported in detail in the Section “Effects of Word Order and Its Interaction with Phonological Overlap” in the Appendix. All effects reported above remained unchanged^4^.

In summary, the results of the disfluency analysis support the hypothesis that phonological onset overlap during unscripted sentence production inhibits phonological encoding. This leads to a preference for delayed production of the overlapping words where grammar permits (Bock, 1987). Our results differ from the majority of previous experiments that were conducted on isolated word production and scripted multi-word production. Many of these studies found facilitatory effects of phonological onset overlap (e.g., Meyer, 1991; Schriefers, 1999; Smith and Wheeldon, 2004; Schnur et al., 2006), although some have found inhibition (e.g., Bock, 1987; Sevald and Dell, 1994; O’Seaghdha and Marin, 2000; Wheeldon, 2003). We return to this point in the Section “General Discussion.” The comparison of our results to previous work is impeded by the fact that most previous work has focused on speech onset latencies and, to a lesser extent, word durations (e.g., Damian, 2003). For this reason, we also analyzed the effect of phonological overlap on speech rates.

## Speech Rates

Just as speech onset latencies provide a measure of the time course of lexical production in isolated word production or highly constrained (e.g., scripted) multi-word utterances, the speech rate before a word provides a continuous measure of processing delays experienced during the phonological encoding of that word: speech rates slow down before points of production difficulty (e.g., Bell et al., 2003).

Hence, if phonological overlap has an inhibitory effect on sentence production, as suggested by the constituent order and disfluency results, we should also observe a slow down in speech rate preceding the sites of phonological overlap. Both perseverative and anticipatory effects are expected, mirroring, and complementing the disfluency results reported in the previous section. Below we test this prediction for the four sentence regions shown in Figure 3.

### Materials and methods

#### Annotation

To gather speech onset latency and speech rate information, all target trials were automatically time aligned using the CMU’s Sphinx Speech Recognition Engines. The package created ELAN annotation files, which were then used to manually check a subset of the automatic alignments. Speech onset latencies from the end of the video to the onset of the first word and four duration measures were extracted: the duration of the head of the subject expression, the verb, and the head of the theme expression. These measures were used to calculate speech rates for the four sentence regions shown in Figure 3. Following previous work, speech rates were calculated in (log-transformed) syllables/s (e.g., Bell et al., 2003). Since we were interested in the speech rates in the different sentence regions, we used the duration of a word as the estimate of the speech rate preceding the next word. For example, if the subject was *Hannah* and its duration was 421 ms, the estimate for the pre-verb speech rate would be 4.75 syllables/s and its log-transformed value would be 1.56. The log inverse of the latency was employed as an estimate of the speech rate preceding the verb, thereby putting all speech rate measures on the same scale.

To assess the reliability of the automatic alignment we examined all 151 trials with detected latencies of less than 350 ms (since those were suspected to be most error-prone) and 40 randomly selected trials each with detected latencies between 350–600, 600–1500, and above 1500 ms. Overall, the automatic aligner returned reliable results. For detected latencies over 600 ms, we found that all trials were correctly annotated. For detected latencies between 350 and 600, we found that the aligner has provided the wrong latency in 3 out of 40 trials (7.5%). We found high error rates only for detected latencies below 350 ms (65%). There was no evidence that alignment errors were in any way dependent on the conditions of the experiment (all χ^2^- and *t*-tests, n.s.). We used the manually corrected latencies for trials for which the aligner had originally detected a latency of less than 350 ms and the automatically detected latencies for all other cases. Based on the above annotation, the remaining alignment error is estimated at only 2%.

The average speech onset latency was 851 ms (SD = 239 ms). The corresponding pre-subject speech rate was 1.27 syllables/s (SD = 0.35 syllables/s). Unsurprisingly, speech rates in the remainder of the sentence were higher (pre-verb: 4.8 syllables/s, SD = 1.45 syllables/s; pre-theme: 5.2 syllables/s, SD = 1.63 syllables/s; pre-recipient: 4.1 syllables/s, SD = 1.31 syllables/s).

#### Analysis

For technical reasons speech rates could not be identified reliably for some participants, resulting in the exclusion of 266 cases (12.9%). We also excluded speech rates that deviated more than 2.5 SD from the mean (after applying a log-transformation), removing another 139 cases (6.7%). This left 1656 cases from 48 participants.

The statistical procedure was identical to that used in the previous sections, except that here we employed a mixed linear, rather than logit, analysis since speech rates are not binomial outcomes (for an introduction to mixed linear models, see Baayen et al., 2008). See the Section “Details on the Random Effect Structure of the Analyses” in the Appendix for details about the random effect structure of the analyses. Results did not depend on the choice of the random effect structure. Below, we report significance values based on *t*-values. While this statistic is known to be anti-conservative, it is familiar to readers and we confirmed all results by means of model comparison and, whenever possible, MCMC sampling.

### Results

**Figure 5:**
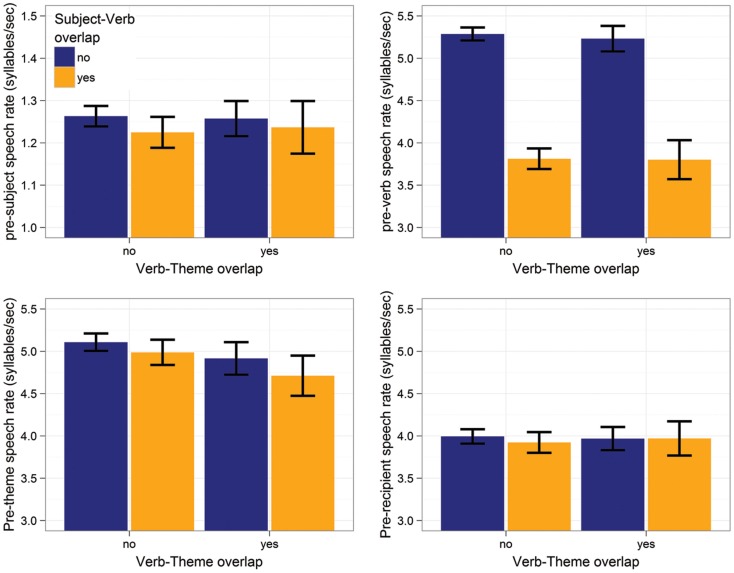
**Effects of subject-verb and verb-theme overlap on speech rate in the pre-subject (top-left), pre-verb (top-right), pre-theme (bottom-left), and pre-recipient region (bottom-right) Error bars give 95% non-parametric confidence interval**.

Table 3 and Figure 5 summarize the results^5^. For the pre-subject region, subject-verb overlap resulted in slower speech rates (*p* < 0.04), whereas verb-theme overlap had no effect (*p* > 0.7). The same pattern is observed for the pre-verb region: only subject-verb overlap resulted in significantly slower speech rates (*p*s < 0.0001). In the theme region, subject-verb overlap had no effect (*p* > 0.7), but verb-theme overlap resulted in significantly slower speech rates (*p* < 0.01). Finally, there were no effects on the pre-recipient region (*p*s > 0.19). The interaction between subject-verb and verb-theme overlap never reached significance (*p*s > 0.4).

**Table 3:** **Speech rate results by sentence region**.

Phonological overlap	Subject-verb			Verb-theme			Subject-verb: verb-theme		
Region	$\hat{\beta }$	*t*	*p*	$\hat{\beta }$	*t*	*p*	$\hat{\beta }$	*t*	*p*
Pre-subject	−**0.03**	−**2.1**	**<0.04**	0.01	0.3	>0.7	−0.02	−0.8	>0.4
Pre-verb	−**0.06**	−**5.3**	**<0.0001**	−0.01	−0.8	>0.4	−0.01	−0.3	>0.7
Pre-theme	−0.01	−0.3	>0.7	−**0.03**	−**1.9**	**<0.07**	0.01	0.2	>0.8
Pre-recipient	0.03	1.3	>0.19	−0.01	−0.2	>0.9	0.01	0.1	>0.9

*Bold indicates the significant effects*.

Since the speech rate over a word is known to be affected by surrounding disfluencies (e.g., Bell et al., 2003), we repeated all analyses while including a control predictor for the presence of disfluencies in the sentence region (the dependent variable of the fluency analysis presented in the previous section). In all cases, the presence of a disfluency was associated with a significant decrease in speech rate and this effect was always of similar magnitude (pre-subject region: $\hat{\beta }$ = − 0.21,*t* = −4.5, *p* < 0.0001; pre-verb: $\hat{\beta }$ = − 0.27, *t* = −10.9 *p* < 0.0001; pre-theme: $\hat{\beta }$ = − 0.22,*t* = −9.0 *p* < 0.0001; pre-recipient: $\hat{\beta }$ = − 0.19, *t* = −6.6 *p* < 0.0001). Crucially, these effects were mostly orthogonal to the effects reported above. Only the marginal effect of verb-theme overlap on the speech rate in the pre-theme region lost significance (*p* = 0.1). All other effects reported in Table 3 remained unchanged.

### Discussion

The speech rate results confirm and extend the results of the fluency analysis. Perseverative effects of both subject-verb and verb-theme overlap were observed in the pre-verb and pre-theme region, respectively.

Unlike in the disfluency analysis, we found anticipatory effects of subject-verb overlap on speech rates in the pre-subject region. This confirmed the explanation offered above: Given that all sentences in the current experiment started with proper names, most speakers preferred to delay the speech onset (here measured in terms of the pre-subject speech rate) rather than to produce a filled pause.

The opposite preference seems to hold once speech is initiated. While we found an anticipatory effect of verb-theme onset on fluency in the pre-verb region, no such effect on speech rate is observed.

Paralleling the *post hoc* analyses presented for fluency above, we conducted additional analyses to assess potential effects of constituent order on speech rate. These are reported in the Section “Effects of Word Order and Its Interaction with Phonological Overlap” in the Appendix. We found no significant interactions of constituent order and phonological overlap. We did, however, find significant main effects of constituent order for both the pre-theme and the pre-recipient region. In both cases, speech rates were higher in the theme-last order, paralleling the result reported above for fluency. This suggests that the theme-last word order was easier to produce for participants in our experiments, regardless of whether any of the expressions in the sentence shared their phonological onsets.

Taken together the fluency and speech rate results suggest that phonological onset overlap between words that are planned in temporal proximity inhibits their phonological encoding. This leads to increased rates of hesitations, filled pauses, and restarts or slowed speech rates or both before the phonologically overlapping words. The fluency and speech rate results provide evidence that phonological onset overlap inhibits phonological encoding, thereby providing a plausible explanation for the observed effect of phonological overlap on constituent order (see also Bock, 1987). Phonological overlap makes it less likely that phonological encoding of the theme has been completed in time for articulation if the theme was produced before the recipient. Speakers avoid the otherwise resulting suspension of speech by first producing the recipient, which does not overlap phonologically with any other word in the sentence. This results in the increased preference for theme-last order in the presence of verb-theme overlap. In short, the productive difficulty resulting from phonological onset overlap can affect grammatical encoding because speakers prefer to order material that is available for articulation earlier (cf. Levelt and Maassen, 1981; Bock, 1987; Ferreira, 1996; Ferreira and Dell, 2000).

## General Discussion

We set out to explore the effects of phonological onset overlap on phonological encoding in unscripted speech, in order to address conflicting results in previous work that employed isolated word production or scripted multi-word production. We found an increased number of disfluencies and slower speech rates before words that shared their phonological onset with other words in the sentence. Additionally, speakers were more likely to produce constituent orders that increased the distance between phonologically overlapping words. In short, the results of all our studies point to phonological onset overlap having an inhibitory effect on phonological encoding.

Our findings confirm the inhibitory effects of phonological onset overlap on constituent order and overall fluency reported in Bock (1987). The results are also compatible with the results of several previous studies that have found inhibition in isolated word naming (O’Seaghdha et al., 1992; Wheeldon, 2003) or scripted multi-word production (Sevald and Dell, 1994). Inhibition for phonological onset overlap is predicted by phonological competition models, such as the parallel-then-sequential phonological competition model (O’Seaghdha and Marin, 2000) and the sequential cueing model (Sevald and Dell, 1994).

The current results also provide further support for the results we reported in Jaeger et al. (2012): we found that speakers seem to have a bias against selecting words that lead to phonological overlap, if there are alternative words with the same or sufficiently similar meanings. These findings were obtained on the same data that was analyzed in the current studies. Recall that target trials were compatible with the three verb lemmas *give*, *hand*, and *pass*. We found that speakers exhibited a weak bias against selecting the verb that shared its onset with the subject expression (*Gabe*, *Hannah*, or *Patty*). Similar inhibitory effects of phonological overlap on lexical selection are also reported in Janssen and López-Pérez (submitted). In Jaeger et al. (2012), we hypothesized that this bias against sequences of phonologically overlapping words might stem from speakers learning to avoid sequences that are difficult to produce (cf. Dell et al., 2008). We reported preliminary evidence that, when speakers produced subject-verb overlap, this led to increased disfluency before the verb. Here, we confirmed and extended this result, while controlling for a large number of potential confounds.

The results of our studies do, however, seem to conflict with a series of other studies on phonological onset overlap (e.g., Meyer, 1991; Meyer and Schriefers, 1991; Schriefers, 1999; Smith and Wheeldon, 2004; Schnur et al., 2006; Yang and Yang, 2008). This conflict is not limited to the current study. Rather, research on isolated word production and the scripted production of word sequences has often returned conflicting results, depending on the specific paradigm. In the remainder of this section, we first discuss possible reasons for this difference in results. We then briefly discuss our results with respect to (a) the scope of phonological encoding during unscripted sentence production and (b) recent proposals that grammatical encoding interacts with phonological encoding. We close with a review of the paradigm employed here.

### What makes phonological onset overlap inhibit phonological encoding?

Despite the rather striking conflict between previous studies, this issue has received surprisingly little attention in the literature. This is not for lack of evidence that paradigm-specific properties affect whether phonological onset overlap results in facilitation or inhibition (e.g., Damian and Martin, 1999; O’Seaghdha and Marin, 2000; Starreveld, 2000). We reviewed over 200 published experiments and annotated them for more than 50 features of the stimuli and the procedure.

For example, we categorized experiments according to whether their phonological primes were verbally comprehended or not and, if yes, whether participants heard or read the prime. We also characterized whether the target was elicited by reading or hearing word or by describing a picture, whether the target was produced in isolation or as part of a larger unit. For multi-word production, we categorized experiments according to whether the context around the target word was only comprehended or produced, whether it was scripted or unscripted, and so on. Other properties we annotated included the phonological and grammatical properties of the prime and target (e.g., the amount of overlap; the number of syllables; the syntactic category) and the overall procedure. Of all these features, only two stood out as being strong predictors of facilitation or inhibition (note though, that for many contrasts, there were too few experiments to determine with certainty whether the feature affected the results). One of these features is when the prime is processed with regard to the target. The other feature is the modality in which the prime is presented and whether the prime is produced or not. We discuss these features in turn.

In one of the most frequently used paradigms, the prime is either orthographically or auditorily presented and hence apprehended linguistically (e.g., word-word interference and picture–word interference, Meyer and Schriefers, 1991; Costa and Caramazza, 2002). In the typical setup, participants are shown line drawings, which they have to name as quickly as possible (the target). On a subset of the trials, however, a distractor word (the prime) is shown in the same screen location at a certain interval before or after the onset of the picture display (the stimulus onset asynchrony, or SOA). When the prime is displayed briefly before, during, or after the target picture, phonologically onset overlapping primes lead to facilitation (e.g., −150 ms < SOAs < 300 ms; e.g., Schriefers et al., 1990; Meyer and Schriefers, 1991).

Interestingly, this facilitatory effect seems to disappear when more time passes between the presentation of the prime and the target (e.g., −500 ms < SOA < −300 ms; e.g., Meyer and Schriefers, 1991; O’Seaghdha et al., 1992). For even longer SOAs between linguistic apprehension of the prime and production of the target (SOA < −650 ms), phonological onset overlap seems to lead to inhibition (Sullivan and Riffel, 1999). The same result is found for other paradigms, in which processing of the prime precedes the production of the prime by at least 650 ms (e.g., Bock, 1987; Sevald and Dell, 1994; Wheeldon, 2003 and our results).

Typical conversational speech proceeds at speech rates of about 3–5 syllables per second. Even in paradigms in which participants are encouraged to talk as fast as possible, repeating the same word sequences over and over again, average per-word durations of about 220–320 ms are observed (Sevald and Dell, 1994; O’Seaghdha and Marin, 2000). Hence, even when two phonologically overlapping words occur adjacent to each other (e.g., as in the case of subject-verb overlap in our experiment), this would correspond to a SOAs that probably are smaller than 320 ms. In other words, the SOAs observed in conversational speech fall into the range where inhibition is the expected result.

These data seem to suggest that inhibition vs. facilitation depends on the relative time course of the phonological processing of the prime and target. The situation is, however, somewhat less clear than it appears. Many of the studies in which longer latencies between prime and target were employed also differ from the typical picture–word interference paradigm in that participants had to *produce* the prime (Bock, 1987; Sevald and Dell, 1994; Sullivan and Riffel, 1999; Wheeldon, 2003 and our results). This raises questions as to whether the inhibitory effects observed in these studies are due to the longer SOAs or due to the fact that speakers produce the prime. Some evidence for the former hypothesis comes from the observation that production of the prime does not necessarily lead to facilitation. Several experiments have investigated phonological overlap when the prime was both linguistically apprehended and produced immediately before the target. Some of these studies found facilitation (Janssen and Caramazza, 2009, Experiment 1b and 2; Smith and Wheeldon, 2004), while other found inhibition (Sullivan and Riffel, 1999; also Janssen and Caramazza, 2009, Experiment 1a).

It is thus tempting to conclude that the primary determinant of facilitation vs. inhibition is *when* the prime is processed relative to the planning of the target – a hypothesis we think deserves further attention in future work. Specifically, it seems that late processing of the prime leads to facilitation, whereas early processing leads to inhibition. This predicts that even picture–word interference paradigms should elicit inhibitory effects for phonological onset overlap if their SOA is sufficiently long.

If confirmed, one possible account of this find would be in terms of competition models (e.g., Sevald and Dell, 1994; O’Seaghdha and Marin, 2000). In these models, the ease of producing a target depends both on its own activation and that of other words. Processing a prime will increase the activation of its phonemes. To the extent that these phonemes are shared with the target, this facilitates the production of the target. However, further activation to the shared phonemes (e.g., as a result of processing the target) is assumed to feedback to the prime and from there to the prime’s phonology, creating competition between the prime’s and the target’s phonology. For onset overlap, these models predict inhibition if the prime has been fully processed before the target (Sevald and Dell, 1994), as observed here. Now consider a scenario in which the prime is processed phonologically after the phonological encoding of the target has already advanced to a point where the target has sufficient activation to not be affected by competition with the prime. At that point, activation from the overlapping phonology might have a purely facilitatory effect. Future simulation studies with competition models are necessary address this possibility.

Next, we discuss the extent to which our results provide insights about the time course of phonological encoding during unscripted language production.

### The time course of phonological encoding during unscripted sentence production

To the best of our knowledge, previous investigations of the scope of phonological planning have been limited to scripted speech. This work has found a relatively small scope of phonological encoding, such as the next syllable (Griffin, 2003) or the current prosodic word (Wheeldon and Lahiri, 1997). Smith and Wheeldon (2004) found faster speech onset latencies for sentences with “The cat and the cap move up” (phonological overlap with the same phrase) compared to “The cat and the rock move up” (no phonological overlap), but not for sentence like “The cat moves above the cap” (phonological overlap across phrases) compared to “The cat moves above the rock” (see also Hilliard et al., 2011; Yang and Yang, 2008). This seems to suggest a slightly larger scope than the current prosodic word. It is, however, worth mentioning that the previous findings stem from paradigms in which responses were highly scripted: responses for which phonological overlap effects were observed always followed the schema “the X and the Y move up/down.” It is thus possible that speakers were able to phonologically encode X and Y at the same time because the intervening words “and the” were always the same. Another explanation is that “and the” became cliticized forming one prosodic word with either X and Y, in which case the result observed by Smith and Wheeldon would provide further evidence that the scope of phonological encoding during sentence production is the prosodic word (Wheeldon and Lahiri, 1997).

Our results are mostly compatible with this hypothesis. We find effects of subject-verb and verb-theme overlap. Since the subject always immediately preceded the verb, this result confirms that phonological encoding of the next (prosodic) word begins before the articulation of the current word is initiated (see also Griffin, 2003). Under the assumption that the indefinite and definite determiners (*a* and *the*, respectively), tend to cliticize to surrounding strong syllables, together forming one prosodic word, Wheeldon and Lahiri’s (1997) proposal would also capture effects of verb-theme overlap in sentence like *Patty* *handed the* *hammer to the boy*.

Perhaps more surprisingly, we found little evidence that the effect of verb-theme overlap depended on word order. For example, the anticipatory effect of verb-theme overlap on the pre-verb speech rate was the same for the theme-first and theme-last order. This suggests that phonological overlap can affect phonological encoding even when the phonologically overlapping word ends up being produced many words later (e.g., decreased fluency and speech rate before the verb for sentences like *Patty* *handed the boy the* *hammer*). We see two possible interpretations of this result. First, this might provide evidence that in unscripted speech, phonological encoding can have a larger scope than in scripted speech. For example, it is possible that speech rates in our experiment were higher than those in scripted speech (no speech rates are reported for Smith and Wheeldon, 2004) and that higher speech rates require more advance phonological planning. Alternatively, it is possible that phonological encoding of the theme begins before articulation of the verb, regardless of the constituent order that is eventually produced.

In this context, it is also interesting that we did not find an interaction of subject-verb and verb-theme overlap on fluency or speech rate in any of the sentence regions. Of particular interest is that we did not find such an interaction for the pre-subject and pre-theme regions. Recall that we restricted our analysis to data for which the subject and theme only had overlapping onsets if both of them overlapped phonologically with the verb. The analyses reported above *ex*cluded all cases in which there was no subject-verb overlap and no verb-theme overlap but subject-theme overlap (e.g., *Hannah gave the boy the* *hammer*, Table 1 above). If phonological overlap between the subject and theme had a detectable effect on production difficulty in any of the sentence regions, this should have been reflected in an interaction of subject-verb and verb-theme overlap. This interaction would have been super-additive if subject-theme overlap had inhibitory effects or sub-additive if subject-theme overlap had a facilitatory effect on phonological encoding. There was no sign of such an interaction (cf. Tables 2 and 3 above). As reported above, we also confirmed that no such interactions emerged once the post-verbal constituent order was controlled for (theme-first vs. theme-last; see results reported in Effects of Word Order and Its Interaction with Phonological Overlap in the Appendix). This suggests that the phonological encoding of the subject and theme did not overlap temporally – or at least not in sufficiently many cases. This finding is compatible with early work by Lindsley (1975, 1976; see also Oppermann et al., 2010). Lindsley employed a picture description paradigm to investigate how much of intransitive or transitive sentences speakers planned before they initiated speech. Based on speech onset latency, Lindsley concluded that the subject and parts of the verb, but not the theme, were planned prior to initiating articulation of the first word of the sentence. Applied to the current data, this suggests that phonological encoding of the theme does not begin until after articulation of the subject has been initiated^6^.

Since, in our data, the speech rates before the theme were comparable to those before the verb (cf. Figure 5), this suggests that the presence of verb-theme overlap effects on production difficulty is *not* due to high speech rates in unscripted speech compared to scripted speech. Rather, at least for the types of sentences investigated here, phonological encoding of the theme seems to overlap temporally with the phonological encoding of the verb.

Such early phonological encoding of the theme even when the speaker eventually chooses the theme-last order might be surprising at first glance. It is theoretically possible that the theme is always – or at least substantially more frequently – encoded before the recipient, regardless of the constituent order eventually produced. For example, it is possible that phonological encoding is triggered in the order in which grammatical functions are assigned during functional processing (cf. Bock and Levelt, 1994). This seems unlikely, however, given that the theme-*last* order is overall more frequent in English (Bresnan et al., 2007) and produced with faster speech rates in our experiment (see Effects of Word Order and Its Interaction with Phonological Overlap in the Appendix). Another possibility is that phonological encoding of the theme and recipient takes place in parallel. This explanation is compatible with race accounts of availability-based production (Ferreira, 1996; Ferreira and Dell, 2000). According to these accounts, speakers select the constituent order that places whichever constituent is ready for articulation first. Although not commonly portrayed as such, this seems to entail at least partially parallel processing of the first words in the two possible constituent orders. Race accounts of availability-based production have received independent support because they correctly predict a variety of cross-linguistically observed properties of grammatical encoding (e.g., Prat-Sala and Branigan, 2000; Ferreira and Yoshita, 2003; Kempen and Harbusch, 2004; Jaeger and Wasow, 2006; Branigan et al., 2008).

We conclude that the results of the current study provide further support for race accounts of grammatical encoding. Further, our results are compatible with the hypothesis that the average scope of advance phonological planning in unscripted speech is one prosodic word (Wheeldon and Lahiri, 1997).

### Is there evidence for an interaction of phonological and grammatical encoding?

In the current experiment, significant inhibitory effects were observed for phonological overlap between nouns and verbs (e.g., subject-verb and verb-theme overlap). This is worth noting because – with very few exceptions – previous work has focused on phonological overlap between nouns (e.g., Bock, 1987; Meyer and Schriefers, 1991; O’Seaghdha et al., 1992; Smith and Wheeldon, 2004). A notable exception is recent work by Janssen and Caramazza (2009; see also Janssen et al., 2008). In a series of experiments, Janssen and Caramazza examined the effect of phonological onset overlap for noun-noun, noun-adjective, adjective-noun, noun-verb, and adjective-adjective-noun sequences in English. Janssen and Caramazza found inhibition (slower speech onset latencies) for phonologically overlapping noun-noun sequences, no effect for noun-adjective sequences, and facilitation for all other types of sequences. They interpret these results to argue (a) that the effect of phonological overlap depends on the grammatical category of the overlapping words, (b) that phonological overlap in words sequences that are “produced in a canonical order” facilitates production (Janssen and Caramazza, 2009, p. 1266), and (c) that grammatical encoding interacts with phonological encoding (see also Abrams and Rodriguez, 2005).

The current experiment does not provide evidence in support for any of (a–c). We found inhibitory effects for both noun-verb and verb-noun overlap. We did not find any evidence for effects of noun-noun overlap (subject-theme overlap). This would have resulted in an interaction between subject-verb and verb-theme overlap, which, however, was not observed. As discussed in the previous section, the most likely explanation for this is that the two phonologically overlapping nouns in our target sentences were too far apart from each other, so that their phonological encoding did not overlap temporally. The next most relevant comparison is the result obtained by Bock (1987). In her experiment, she observed the same inhibitory effect on fluency and constituent order for noun-noun overlap that was observed here for noun-verb and verb-noun overlap. We conclude that the available data from unscripted language production does not provide evidence for an interaction of grammatical and phonological encoding in the sense outlined by Janssen and Caramazza (2009).

One possible explanation for the results obtained by Janssen and Caramazza (2009) is that the differences in the effects of phonological overlap that they observed are due to differences in the average speech rate with which the different word sequences were produced. A review of their experiments reveals that their studies did not only differ with regard to the type and order of grammatical categories. For example, noun-noun and noun-verb sequences were elicited “by a picture object with a superimposed word” (Janssen and Caramazza, 2009, p. 1263). Successful completion of this task thus required object recognition and word recognition. Adjective-noun and noun-adjective sequences, on the other hand, were elicited “by a word in a certain ink color” (Janssen and Caramazza, 2009). Successful completion of this task required color recognition and word recognition. The two tasks also differ in that the former involves a word and an object, whereas the latter involves just an object. These differences might have affected to what extent the phonological encoding of the two words overlapped temporally or what *aspects* of phonological encoding overlapped temporally.

Whatever the reasons are, the current results do not support Janssen and Caramazza’s conclusion that a lemma’s grammatical category and phonological encoding interact. Additionally, we find exactly the opposite of what Janssen and Caramazza propose: in the production of canonical sequences, phonological onset overlap seems to lead to inhibition, not facilitation.

## Conclusion

Work on phonological encoding has primarily investigated isolated word production. However, in natural language production, words are produced as part of a message. That is, lexical production takes place in the presence of other production processes (e.g., grammatical encoding). This raises the question whether findings from isolated word production transfer to production in a sentential context. To the extent that lexical production as part of larger units (e.g., a phrase or sentence) has been studied, this has usually been achieved by scripting production. These scripted production paradigms have at least three potential problems for research on phonological encoding. First, most of these paradigms involve verbal apprehension that immediately precedes or temporally overlaps with production of the relevant target words. This, as we have discussed above, might affect how much results from the paradigms reflect processes that are genuine to production rather than to the interplay between production and comprehension. Second, scripted and highly repetitive speech might invite strategic planning of a type that is not typical to everyday language production. It is conceivable that this affects the scope of phonological planning. Third, scripted production paradigms have often elicited word sequences that did not form complete utterances (e.g., Schriefers, 1999; Janssen and Caramazza, 2009; but see Smith and Wheeldon, 2004; Yang and Yang, 2008; Hilliard et al., 2011).

For these reasons, we extended a paradigm first introduced by Bock (1987) to study phonological encoding during unscripted sentence production. The results from this paradigm provide further support for findings that the effects of phonological overlap on phonological encoding depend on specifics of the paradigm (cf. O’Seaghdha and Marin, 2000; Starreveld, 2000). Contrary to a large number of studies within the picture–word and picture–picture interference paradigm, but in line with studies that avoid verbal apprehension during or immediately preceding production, we find that phonologically overlap between onsets of adjacent or proximate words generally leads to inhibition (e.g., more disfluencies, slower speech rates).

## Conflict of Interest Statement

The authors declare that the research was conducted in the absence of any commercial or financial relationships that could be construed as a potential conflict of interest.

## Footnotes

^1^Onset and rhyme overlap have been shown to differ in their effect on phonological encoding (e.g., O’Seaghdha and Marin, 2000; Sevald and Dell, 1994; see also Jaeger et al., 2012 compared to Rapp and Samuel, 2002). Below, we use the shorter term phonological overlap to refer to phonological onset overlap.

^2^These cases should have been removed for the analyses reported in Jaeger et al. (2012). Their removal does not, however, change the results reported in Jaeger et al. (2012).

^3^A model with all five conditions led to high collinearity between the conditions due to the relatively small number of cases in the subject-verb-them overlap condition compared to the subject-theme overlap condition.

^4^Interestingly, we also found that theme-last order was associated with fewer disfluencies in the pre-recipient regions. This might be taken as further evidence that theme-last was a default choice of participants in our experiment.

^5^We note that Figure 5 over-represents the effect size of subject-verb overlap on speech rates in the pre-verb region. A look at Table 3 shows that the effect size in pre-verb region was twice as large as in the pre-subject region and not many-times larger (as Figure 5 would suggest). Note that the model controlled for potential confounds (see Overview over the Statistical Approach and Overview over Control Predictors in the Appendix), whereas the figures do not.

^6^More precisely, it is sufficient to assume that the aspects of phonological encoding that result in inhibition when they overlap temporally between two words that share the same phonological onset have not been initiated for the verb before the relevant aspects of phonological encoding for the subject have been completed. See also the previous section.

## Appendix

### Overview over the statistical approach

We investigated the effects of phonological overlap by means of mixed effect regression models and principal component analysis.

Specifically, the use of these regression methods, allowed us to control for a larger number of potential confounds. These control predictors are introduced in the Section “Overview over Control Predictors”. We employed principal component analysis as a principled way to determine a set of (orthogonal) control predictors that was (a) sufficiently small to reduce problems due to overfitting and power loss while at the same time (b) capturing the effects of a much larger set of potential confounds. This procedure is described in the Section “Controlling for Potential Confounds.” We used mixed regression models and the use of model comparison to effectively control for statistical dependencies due to repeated measures from the same participants, despite the unbalance nature of unscripted language data like ours. The random effect structure of these models was determined by model comparison in order to strike an effective balance between need for statistical conservativeness with the constraints imposed on the analyses due to data sparsity and data imbalance. This procedure is described in the Section “Details on the Random Effect Structure of the Analyses.” Finally, we conducted additional auxiliary analyses that are reported in the Section “Effects of Word Order and Its Interaction with Phonological Overlap.”

None of these procedures is perfect (see, e.g., Harrell, 2001 on stepwise model reduction, which is known to be anti-conservative; see Baayen, 2007 for potential problems with principal component analysis; see Barr et al., 2011 and Jaeger et al., 2011 for discussions of how to chose the random effect structure of mixed models). Our choices reflect a best effort to strike a reasonable balance between three conflicting goals: the desire to be statistically conservative, thereby increasing the likelihood of replicability; the desire to gain insights about the data; and the desire to allow others to replicate our procedure (rather than results).

Fortunately, for this particular data case, the qualitative results do not depend on any of the analysis choices we made. For example, even if the stepwise model reduction described in the Section “Controlling for Potential Confounds” is not applied, the results do not change. Similarly, all results reported here hold under any of the random effect structures considered (as described in Details on the Random Effect Structure of the Analyses).

### Overview over control predictors

The following control predictors were coded for the (head of the) subject, verb, theme, and recipient expressions: (i) frequency with which the word’s first syllable occurs as first syllable of any word of English, (ii) frequency with which the word’s first syllable occurs in any position within any word of English, (iii) frequency of the word, (iv) number of neighbors in the English lexicon with 1-edit distance, (v) frequency-weighted neighborhood density, (vi) mean biphone transitional probability in word, (vii) lexical repetition from previous target trial, (viii) repetition of syntactic structure from previous target trial, (ix) the joint frequency of the subject and verb, (x) the joint frequency of the verb and theme, and (xi) plausibility of meaning expressed in sentence.

Controls (i–vii) capture properties that are known – or have been hypothesized – to affect the time course of lexical production. There properties are thus expected to affect the fluency immediately preceding the word, as the difficulty with lexical retrieval or phonological encoding of upcoming material increases the probability of slowed speech rate, filled or unfilled pauses, and word repetitions (e.g., Shriberg and Stolcke, 1996; Fox Tree and Clark, 1997; Clark and Wasow, 1998; Clark and Fox Tree, 2002). Controls (viii–xi) capture general sentence properties that are hypothesized to affect fluency. Carbary and Tanenhaus (2011) found that syntactic repetition leads to increased fluency in interactive task-oriented dialogues. Similarly, frequent co-occurrence has been found to be associated with higher fluency (Shriberg and Stolcke, 1996). Finally, while we are not aware of studies that investigate the effect of plausibility on fluency, plausibility was included as a control because it has been found to affect a wide variety of behaviors in language processing.

Syllables and lexical frequencies were obtained from CELEX (Baayen et al., 1995). Neighborhood density measures were obtained from the online neighborhood density calculator provided by the Speech and Hearing Lab at Washington University^1^. Joint frequency counts of the subject and verb or verb and theme, respectively, occurring within four words of each other were obtained from the 400 million word Contemporary Corpus of American English (Davies, 2010).

Plausibility norms were obtained using Amazon’s online platform Mechanical Turk^2^. Participants were instructed to rate the plausibility of sentences on a scale from 1 (implausible) to 7 (plausible). Participants were paid 1 cent per sentence. Stimuli were displayed in sets of eight, consisting of three target sentences and five filler sentences from the experiment. Since there was no reason to assume that the subject characters (*Simon*, *Gabe*, *Hannah*, and *Patty*) would affect the plausibility of the physical transfer events in our experiment, all target and filler sentences used *Simon* as the subject. Across participants, target sentences were rated both in the NPNP and NPPP order and with all three possible verbs. Participants saw a verb-theme pairing exactly once. Each stimulus was judged by six participants.

Table A3 summarizes the means of all controls by condition (for details about the five conditions reported in the table, see the main text). The final column contains the results of an ANOVA comparing the control’s mean across only the four conditions that were included in the analyses reported in the main text (subject-verb overlap, verb-theme overlap, subject-theme overlap, and no overlap). For each comparison, we aggregated the data so that there was one data point for each unique value of the relevant grouping variable that was actually observed in the experiment. For example, for properties of the verb (e.g., verb frequency), we obtained the mean frequency for each of the six possible verb forms in each of the five conditions. For the co-occurrence frequency of verb and theme, we obtained the mean values for each condition and each unique combination of verb and (head of) theme observed in the experiment. This corresponds to the standard (though potentially problematic) practice of assessing confounding factors through over the items, rather than the full data set. All frequency, probability, and neighborhood density controls, (i–vi) and (ix–x), were log-transformed before comparing their means across conditions.

As shown in Table A3, several properties of the subject, theme, and recipient head noun, as well as collocational measures and plausibility differed significantly between the phonological overlap conditions. Section “Controlling for Potential Confounds” describes how our modeling procedure addressed the potential of confounds.

### Controlling for potential confounds

The lexical heterogeneity and imbalance of our data set required us to control for potentially confounding effects of the properties described in the Section “Overview over Control Predictors.” When approaching this problem, there are two conflicting considerations. On the one hand, it is desirable to include as many control variables in the analysis as possible. For example, even if a control predictor does not differ significantly across conditions according to the tests given in Table A3 in the Section “Overview over Control Predictors,” it is still possible that it does affect the outcome of interest. More crucially, it also still possible that the control predictor is confounded with the conditions of outcome *once other control predictors are taken into consideration*. On the other hand, the inclusion of too many control predictors in the analysis risks spurious effects due to overfitting and hence increases in the Type I error rate. At the same time, the inclusion of additional control predictors often results in a loss of power and hence an increased Type II error rate. These two problems (overfitting and power) are exaggerated when the corpus at hand is relatively small, as is the case here.

Following standard power considerations for regression analyses (see Jaeger, 2011 and references therein), some of our analyses contained sufficient data for only 5–8 degrees of freedom. Since the design factors alone contribute 4–13 degrees of freedom, depending on the random effect structure (see Details on the Random Effect Structure of the Analyses), the inclusion of all control predictors from Table A3 is not an option.

Based on these considerations, we present a different approach. For each data set we analyze in the main text, we employed principal component analysis (PCA) to determine the most important components of all control predictors listed in Table A3. PCA determines the orthogonal (non-correlated) dimensions of all control predictors. In all cases, the 10 most important of these components cumulatively accounted for at least 96% of the variance among control predictors.

These 10 components were entered into the main analysis together with the 2-by-2 design factors and a random by-participant intercept, coding the four phonological overlap conditions (all centered). Next, stepwise model reduction was performed. At each step, the least significant control predictor was removed from the model as long as *p* > 0.3. This procedure was repeated until no control predictor remained in the model or no control predictor that still was in the model was less significant than *p* = 0.3. The resulting model was the input to the procedure described in the Section “Details on the Random Effect Structure of the Analyses.”

It should be noted that stepwise model reduction is often problematic since it is known to over-estimate the effect of the remaining predictors and to inflate the Type I error rate (Harrell, 2001). Here, the alternative would have been to overfit the data (this was confirmed by bootstrap for a random subset of the analyses), which is at least equally undesirable. More importantly, none of the results reported above changed qualitatively if more control components were included. For many of the analyses reported in the main text, we also conducted additional analyses on (a) subsets of the data or (b) with the original control predictors rather than principal components. The qualitative result patterns remained the same. In short, we report the results of the procedure described here because we believe an adequate and robust solution to the trade-off between the many different challenges inherent to the analysis of unbalanced and heterogeneous data.

### Details on the random effect structure of the analyses

To determine the random effect structure that warranted by the data and yet as parsimonious as possible in order to avoid overfitting, we follow the procedure outlined by Baayen et al. (2008)^3^. The smallest model that was considered (with only a random by-subject intercept) had 7 degrees of freedom. The largest model (with full factorial random by-subject slopes) had 16 degrees of freedom, plus the number of remaining control components from the procedure described in the Section “Controlling for Potential Confounds.”

#### Constituent order

The results reported in the main text held regardless of whether the maximal random effect structure was used or only random intercepts by participant. *A priori* considerations suggested a maximum of 52–79 degrees of freedom (cf. references in Jaeger, 2011), so that overfitting was not a concern. Model comparison did not provide evidence that any random by-subject slopes were required (random intercept-only vs. full factorial model:$\chi_{\Delta \left(\text{dev}\right)}^{2}\left(9\right)$ = 2.1,*p* > 0.9).

#### Disfluencies

All of the results reported here and above also hold if participants that never exhibited disfluencies in the analyzed region are excluded. In this alternative analysis, some of the effects reported above do, however, get stronger. Put differently, the results reported in the main text are conservative estimates. The results reported in the main text held regardless of whether the maximal random effect structure was used or only random intercepts by participant.

##### Pre-subject

*A priori* considerations suggested a maximum of 5–8 degrees of freedom. Model comparison did not provide evidence that any random by-subject slopes were required (random intercept-only vs. full factorial model: $\chi_{\Delta \left(\text{dev}\right)}^{2}\left(9\right)$ = 4.8,*p* > 0.8).

##### Pre-verb

*A priori* considerations suggested a maximum of 6–9 degrees of freedom. Model comparison did not provide evidence that any random by-subject slopes were required (random intercept-only vs. full factorial model: $\chi_{\Delta \left(\text{dev}\right)}^{2}\left(9\right)$ = 3.3,*p* > 0.9).

**Table A1:** **Interactions between post-verbal constituent order and phonological overlap on fluency by sentence region**.

Effect	Theme-last			Theme-last: subject-verb			Theme-last: verb-theme			Theme-last: subject-verb: verb-theme		
Region	$\hat{\beta }$	*z*	*p*	$\hat{\beta }$	*z*	*p*	$\hat{\beta }$	*z*	*p*	$\hat{\beta }$	*z*	*p*
Pre-subject	−0.53	−1.5	>0.14	0.97	1.7	<0.09	0.21	0.3	>0.7	0.70	0.5	>0.6
Pre-verb	−0.47	−1.3	>0.19	**1.26**	**2.33**	**<0.02**	0.13	0.3	>0.8	−0.35	−0.3	>0.7
Pre-theme	0.33	1.6	>0.1	0.13	0.4	>0.6	0.40	1.1	>0.2	−1.40	−1.9	<0.06
Pre-recipient	−**0.67**	−**2.9**	**<0.004**	0.54	1.5	>0.13	−0.80	−1.8	<0.08	−0.09	−0.1	>0.9

*Bold indicates the significant effects*.

As reported in the main text, both main effects of subject-verb and verb-theme overlap reach significance in the intercept-only model (the one preferred by model comparison). In the model with the full factorial random by-subject slopes, only subject-verb overlap reaches significance.

##### Pre-theme

*A priori* considerations suggested a maximum of 15–23 degrees of freedom. Model comparison did not provide evidence that any random by-subject slopes were required (random intercept-only vs. full factorial model: $\chi_{\Delta \left(\text{dev}\right)}^{2}\left(9\right)$ = 2.9,*p* > 0.9).

##### Pre-recipient

*A priori* considerations suggested a maximum of 15–23 degrees of freedom. Model comparison did not provide evidence that any random by-subject slopes were required (random intercept-only vs. full factorial model: $\chi_{\Delta \left(\text{dev}\right)}^{2}\left(9\right)$ = 5.1,*p* > 0.8).

As reported in the main text, there were no significant main effects of subject-verb or verb-theme overlap in the intercept-only model (the one preferred by model comparison). Neither was there a significant interaction between the two factors. There were, however, interactions with constituent order (significant for subject-theme overlap; marginal for verb-theme overlap).

In the model with the full factorial random by-subject slopes, there was a marginally significant inhibitory main effect of subject-verb overlap ($\hat{\beta }$ = 0.30, *z* = 1.7, *p* < 0.1). Subject-verb overlap also interacted with verb-theme overlap, so that the inhibitory effect was further strengthened ($\hat{\beta }$ = 0.85, *z* = 1.8, *p* < 0.08). Additionally, the two interactions with constituent order also observed in the intercept-only model remained significant in the same direction as before (for subject-verb, *p* < 0.05; for verb-theme overlap, *p* < 0.07).

#### Speech rate

*A priori* power considerations suggested a maximum of over 80 degrees of freedom for all of the analyses, so that overfitting was not a concern. Only for the pre-recipient region did changes in the random effect structure lead to different results (see below).

##### Pre-subject

Model comparison did not provide evidence that any random by-subject slopes were required (random intercept-only vs. full factorial model: $\chi_{\Delta \left(\text{dev}\right)}^{2}\left(9\right)$ = 1.6,*p* > 0.9).

##### Pre-verb

Model comparison found that full factorial random slopes should be included in the analysis (random intercept-only vs. full factorial model: $\chi_{\Delta \left(\text{dev}\right)}^{2}\left(9\right)$ = 29.8,*p* < 0.0001). This is the analysis reported in the main text.

##### Pre-theme

Model comparison did not provide evidence that any random by-subject slopes were required (random intercept-only vs. full factorial model: $\chi_{\Delta \left(\text{dev}\right)}^{2}\left(9\right)$ = 6.2,*p* > 0.7; additional tests confirmed that no single random slope led to significant improvements).

##### Pre-recipient

Model comparison did not provide evidence that any random by-subject slopes were required (random intercept-only vs. full factorial model: $\chi_{\Delta \left(\text{dev}\right)}^{2}\left(9\right)$ = 1.6,*p* > 0.9.)

As reported in the main text, there were no significant main effects of subject-verb or verb-theme overlap in the intercept-only model (the one preferred by model comparison). Neither was there a significant interaction between the two factors.

In the model with the full factorial random by-subject slopes, there was a marginally significant inhibitory main effect of subject-verb overlap $\hat{\beta }$ = 0.30,*t* = 1.7, *p* < 0.1. Subject-verb overlap also interacted with verb-theme overlap, so that the inhibitory effect was further strengthened $\hat{\beta }$ = 0.85,*t* = 1.8, *p* < 0.08. That is, the results reported in the main text are conservative in that we found further inhibitory effects if the full factorial random effect structure was used.

### Effects of word order and its interaction with phonological overlap

As mentioned in the main text, we also conducted further analyses that assessed the effect of word order and its interaction with phonological overlap on fluency and speech rates throughout the sentence. To that end, we repeated the analyses for fluency and speech rates over all sentence regions while including an interaction of word order with (i) subject-verb overlap, (ii) verb-theme overlap, and (iii) interaction. Below, we report the results of the interaction. The principal component control predictors and the random effect structure of these auxiliary analyses were the same as reported for the original analyses in the main text.

We first report the results for distribution of disfluencies across the four sentence regions and then the results of the speech rate analyses for the same four sentence regions. The interactions tests were not planned comparisons. To avoid inflated Type I error rates we applied the Bonferroni correction to our assessment of significance. Below, we consider *p* < 0.0127 as significant and *p* < 0.0260 as marginally significant.

**Table A2:** **Interactions between post-verbal constituent order and phonological overlap on speech rate by sentence region**.

Effect	Theme-last			Theme-last: subject-verb			Theme-last: verb-theme			Theme-last: subject-verb: verb-theme		
Region	$\hat{\beta }$	*t*	*p*	$\hat{\beta }$	*t*	*p*	$\hat{\beta }$	*t*	*p*	$\hat{\beta }$	*t*	*p*
Pre-subject	−0.01	−0.1		−0.02	−0.8		−0.04	−1.5		0.04	0.7	
Pre-verb	−0.01	−0.5		0.01	0.2		0.03	1.4		0.02	0.5	
Pre-theme	**0.22**	**12.2**	**<0.0001**	0.01	0.1		−0.01	−0.5		−0.04	−0.6	
Pre-recipient	**0.20**	**9.9**	**<0.0001**	0.01	0.1		−0.04	−1.3		0.09	1.3	

*The only significant effects were found for the pre-theme and pre-recipient region. For both regions, speech rates were higher for the theme-last order, paralleling the effect found for fluency in the pre-recipient region (i.e., fewer disfluencies in the theme-last order). Bold indicates the significant effects*.

**Table A3:** **Means and SD of control predictors in the five conditions summarized in Table 1**.

Phonological overlap	Subject-verb	Subject-verb-theme	Verb-theme	None	Subject-theme	ANOVAs
Measure	*N* = 575	*N* = 175	*N* = 352	*N* = 959	*N* = 369	*F*(3, 198)
**HEAD OF SUBJECT EXPRESSION**						
(Log) frequency of 1st syllable, 1st position	1.92 (0.29)	1.92 (0.29)	2.47 (0.36)	2.47 (0.36)	2.48 (0.36)	207.1***
(Log) frequency of 1st syllable, any position	2.35 (0.11)	2.35 (0.12)	2.64 (0.32)	2.66 (0.33)	2.65 (0.32)	283.5***
(Log) frequency of word form	3.40 (0.16)	3.39 (0.16)	3.64 (0.14)	3.63 (0.14)	3.64 (0.13)	130.8***
(Log) neighborhood density	0.93 (0.37)	0.96 (0.36)	0.85 (0.46)	0.89 (0.45)	0.85 (0.46)	3.5*
**VERB**						
(Log) frequency of 1st syllable, 1st position	5.95 (0.35)	5.93 (0.36)	5.92 (0.38)	5.94 (0.36)	5.95 (0.34)	0.2
(Log) frequency of 1st syllable, any position	5.98 (0.36)	5.96 (0.37)	5.95 (0.40)	5.98 (0.38)	5.98 (0.36)	0.2
(Log) frequency of word form	10.54 (0.99)	10.60 (0.92)	10.55 (0.94)	10.50 (1.01)	10.57 (0.98)	0.4
(Log) neighborhood density	2.54 (0.33)	2.56 (0.33)	2.55 (0.34)	2.54 (0.33)	2.54 (0.33)	0.1
Lexical repetition of most recent target verb	0.82 (0.38)	0.83 (0.38)	0.76 (0.43)	0.76 (0.43)	0.76 (0.43)	2.1
**HEAD OF THEME EXPRESSION**						
(Log) frequency of 1st syllable, 1st position	1.70 (0.72)	1.96 (0.63)	2.01 (0.62)	1.68 (0.82)	1.82 (0.61)	14.5***
(Log) frequency of 1st syllable, any position	3.71 (1.60)	4.01 (1.71)	4.16 (1.67)	3.62 (1.82)	3.99 (1.38)	45.2***
(Log) frequency of word form	3.73 (0.59)	3.64 (0.44)	4.16 (1.67)	3.62 (1.83)	3.79 (0.52)	4.8**
(Log) neighborhood density	0.89 (0.52)	0.96 (0.46)	0.94 (0.49)	0.76 (0.55)	1.04 (0.45)	24.7***
**HEAD OF RECIPIENT EXPRESSION**						
(Log) frequency of 1st syllable, 1st position	2.31 (0.18)	2.34 (0.17)	2.34 (0.19)	2.32 (0.18)	2.32 (0.17)	2.0
(Log) frequency of 1st syllable, any position	2.33 (0.18)	2.36 (0.17)	2.36 (0.18)	2.34 (0.17)	2.34 (0.17)	2.6+
(Log) frequency of word form	5.02 (0.21)	5.00 (0.19)	5.00 (0.19)	5.01 (0.21)	5.02 (0.20)	0.8
(Log) neighborhood density	0.95 (0.51)	1.05 (0.51)	1.04 (0.51)	1.00 (0.51)	0.98 (0.51)	5.1**
**SENTENCE-WIDE PROPERTIES**						
(Log) joint frequency of subject and verb	0.62 (0.20)	0.62 (0.18)	0.79 (0.44)	0.79 (0.44)	0.83 (0.44)	9.5***
(Log) joint frequency of verb and theme	0.99 (0.51)	0.85 (0.38)	0.89 (0.37)	0.94 (0.53)	1.00 (0.48)	6.4***
*(z*-Score) plausibility rating	0.11 (0.96)	−0.48 (1.07)	−0.47 (1.14)	0.23 (0.90)	−0.05 (0.84)	57.7***
Syntactic repetition of most recent target structure	0.80 (0.40)	0.84 (0.37)	0.80 (0.40)	0.82 (0.39)	0.84 (0.36)	0.7

*Note that the subject-theme overlap condition was excluded from the main analysis as described in the Section “Overview of Analyses.” The last column gives the *p*-value based on an ANOVA over only the four conditions included in the main analyses (see text for details). **p* < 0.05; ***p* < 0.01; *** *p* < 0.001*.

#### Fluency

For phonological overlap and the constituent order effects, collinearity was not an issue in any of the analyses (all fixed effect correlation *r*s < 0.3). In all cases, the effects reported in the main text remained unchanged by the inclusion of the constituent order and its interaction with phonological overlap in the analysis. Table A1 summarizes the main effect of post-verbal constituent order and its interaction with the phonological overlap predictors.

We found two significant effects of constituent order (after applying the Bonferroni correction). The first effect, a marginally significant interaction between constituent order and subject-verb overlap is to be interpreted with caution. Recall that *a priori* power considerations suggest that no more than 8 parameters should be employed in analyzing the pre-subject region, and no more than 9 parameters for the pre-verb region (cf. Details on the Random Effect Structure of the Analyses). With the word order interactions, the analysis of the pre-subject region contained eight parameters. The analysis of the pre-verb region contained 13 parameters (see the differences are due to the number of principal component control predictors that were left in the model after applying the procedure outlined in Controlling for Potential Confounds). That is, the analyses of both the pre-subject and pre-verb region are likely unreliable (cross-validation confirmed this). We therefore do not discuss the marginally significant interaction of constituent order and subject-verb overlap further.

Overfitting was not likely to be an issue for the two remaining sentence regions (see Details on the Random Effect Structure of the Analyses). We found a significant main effect of constituent order on fluency in the pre-recipient region, so that speakers were less likely to produce disfluencies before the recipient when the recipient was produced earlier (theme-last order).

#### Speech rate

For phonological overlap and the constituent order effects, collinearity was not an issue in any of the analyses (all fixed effect correlation *r*s < 0.2). In all cases, the effects reported in the main text remained unchanged by the inclusion of the constituent order and its interaction with phonological overlap in the analysis. Table A2 summarizes the main effect of post-verbal constituent order and its interaction with the phonological overlap predictors.

^1^Website can be accessed at http://128.252.27.56/Neighborhood/NeighborHome.asp ^2^Website can be accessed at http://www.mturk.com/mturk/welcome ^3^See also http://hlplab.wordpress.com/2011/06/25/more-on-random-slopes/
